# Supplementary material for: Earthquake nucleation in the lower crust by local stress amplification
Source: Nat Commun. 2020 Mar 12;11:1322. doi: 10.1038/s41467-020-15150-x (PMC7067852; doi:10.1038/s41467-020-15150-x)
Supplement: Supplementary file 1 — Supplementary Information [file 41467_2020_15150_MOESM1_ESM.docx]

Supplementary Information for

**Earthquake nucleation in the lower crust by local stress amplification**

by Campbell et al.

Supplementary Figures


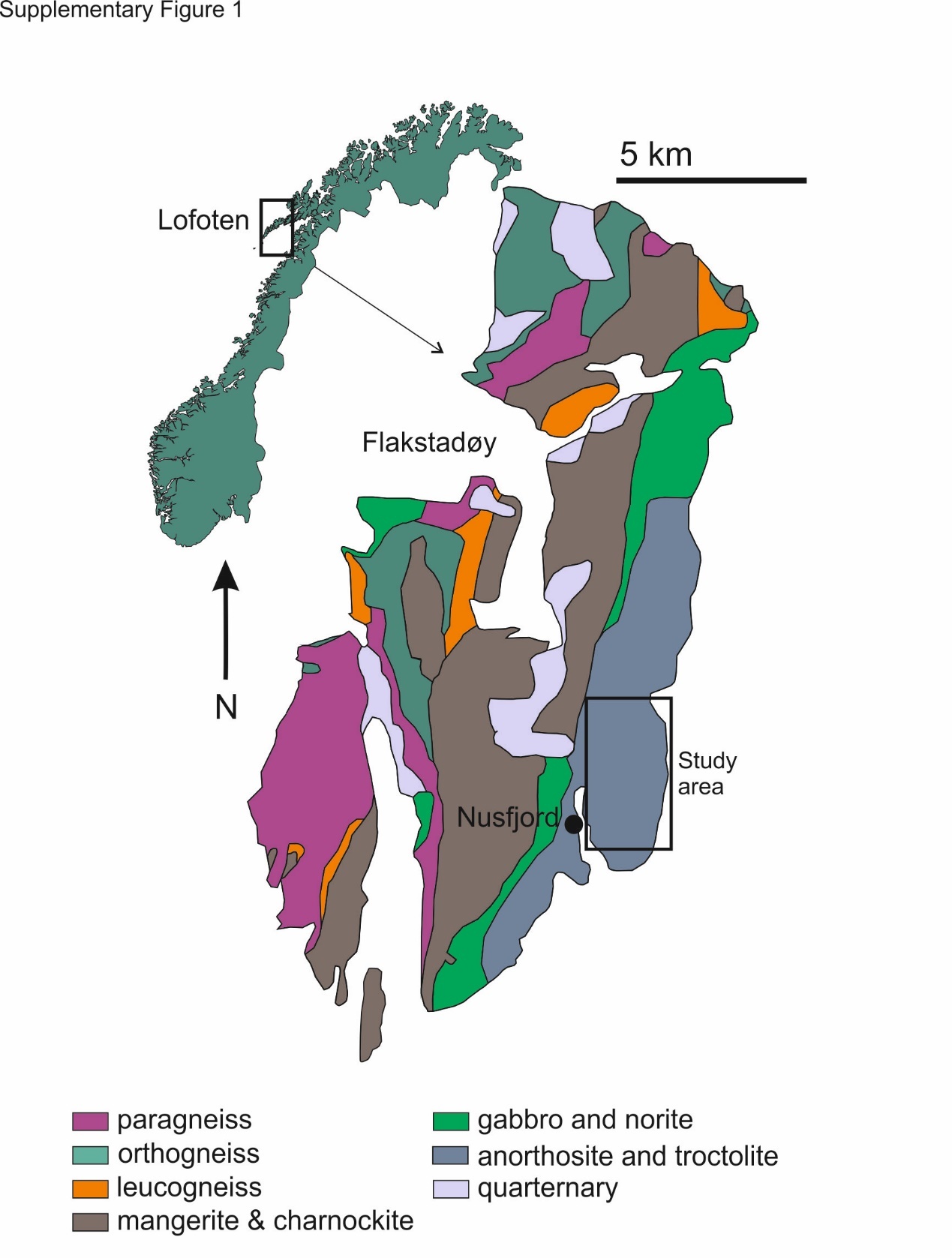


**Supplementary Figure 1:** Location and geological map of Flakstadøy, Lofoten, Norway. Adapted after ref.^1^


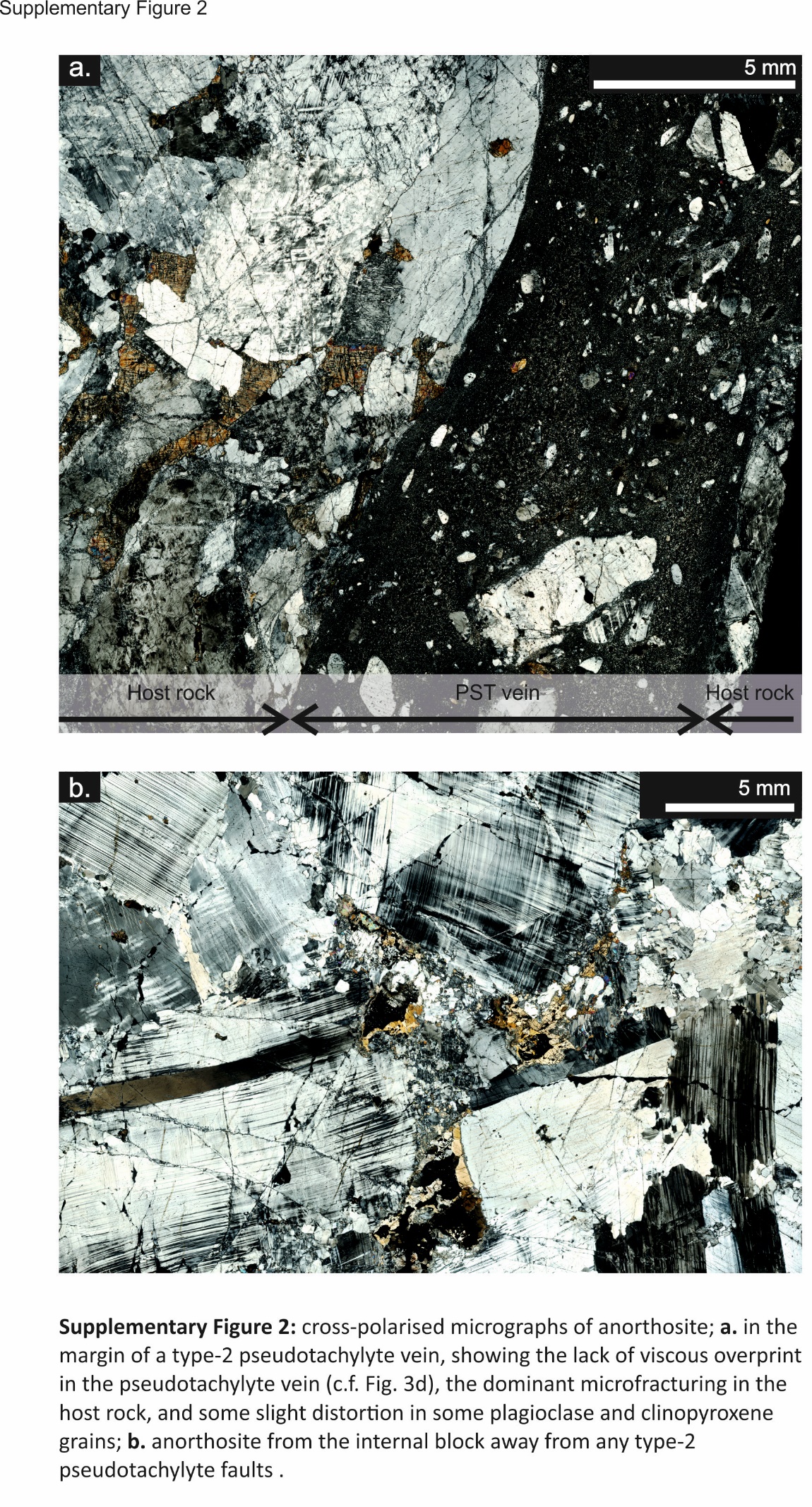


**Supplementary Figure 2:** cross-polarised micrographs of anorthosite; **a**. in the margin of a type-2 pseudotachylyte vein, showing the lack of viscous overprint in the pseudotachylyte vein (c.f. Fig. 3d), the dominant microfracturing in the host rock, and some slight distortion in some plagioclase and clinopyroxene grains [68.0552°N 13.3678°E]; **b.** anorthosite from the internal block away from any type-2 pseudotachylyte faults [68.0557° N 13.3744°E].


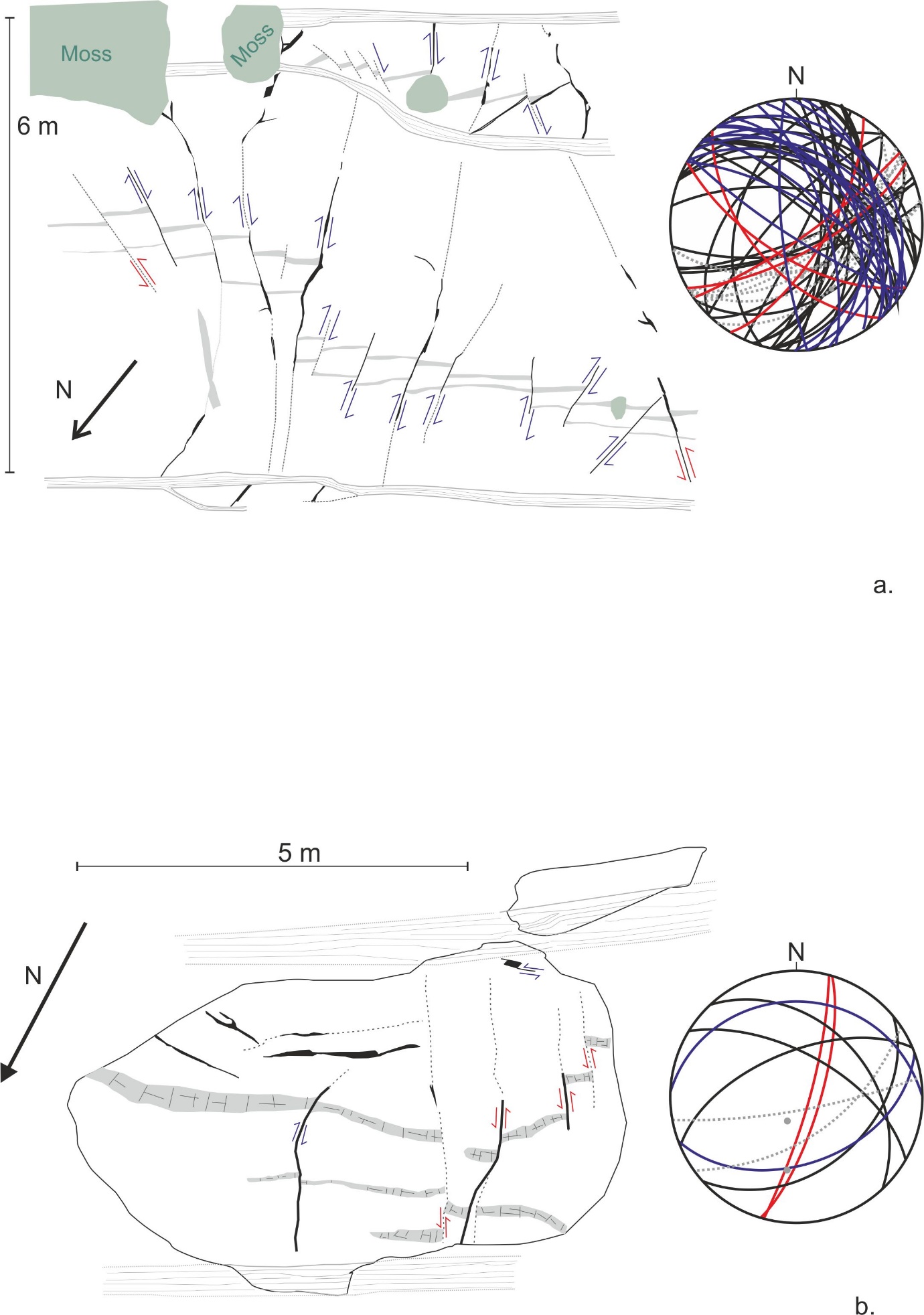


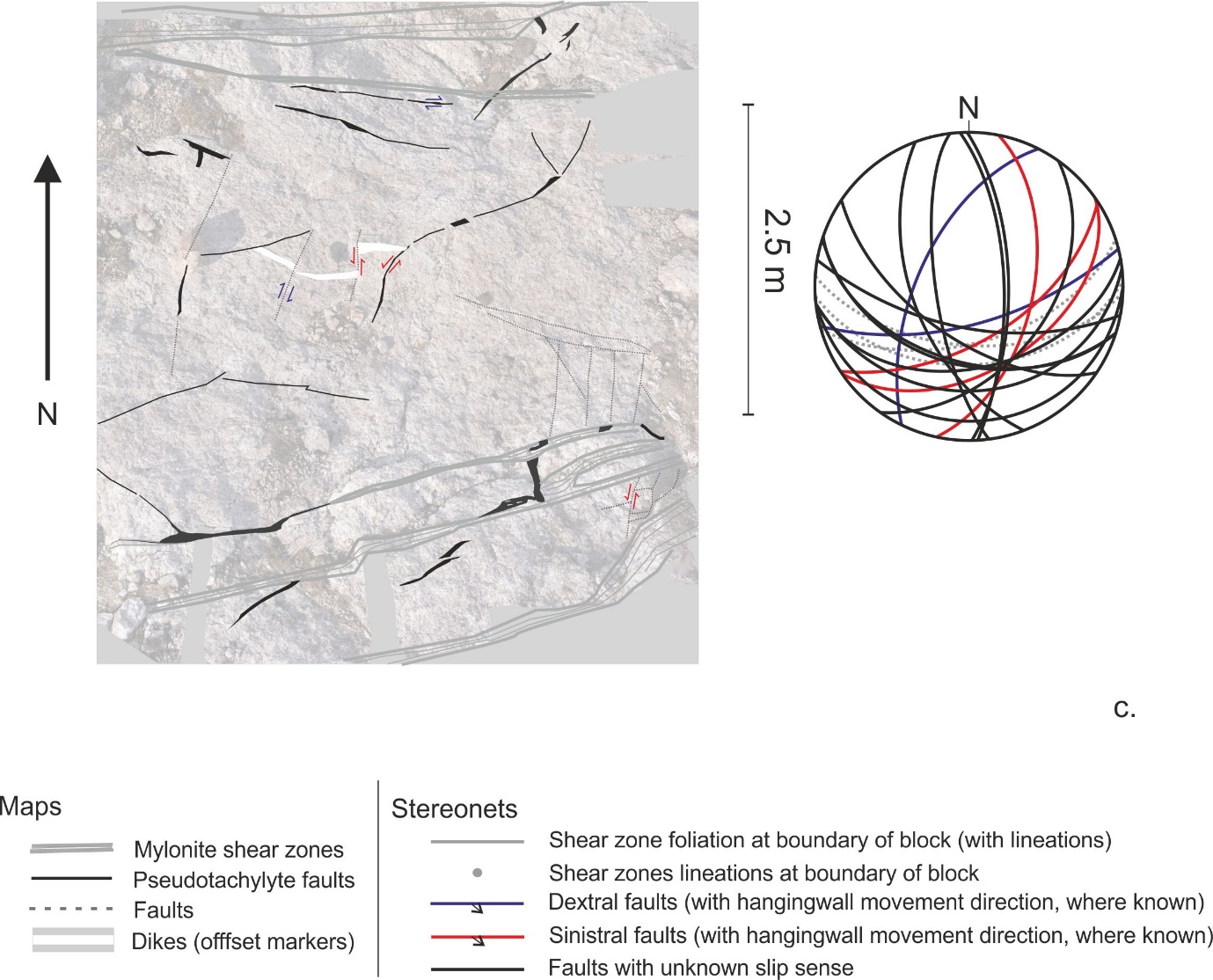


**Supplementary Figure 3:** Sketch maps and stereonets of additional examples of shear zone bounded fault blocks; **a.** Block bounded by *set 1* mylonitic shear zones, located ~100m northwest if the example shown in Fig. 3a [68.0552°N 13.3721°W]. Here, an intermediate shear zone divides the upper and lower pseudotachylyte fault systems; **b.** Block bounded by two *set 1* shear zones, internal pseudotachylytes cut and offset mafic dykes [68.0558°N 13.3614°E]; **c.** Multi-stranded *set 1* shear zone displays pseudotachylyte-bearing faults < 1m in length between the individual, anastomosing mylonite branches [68.0518°N 13.3705°E].


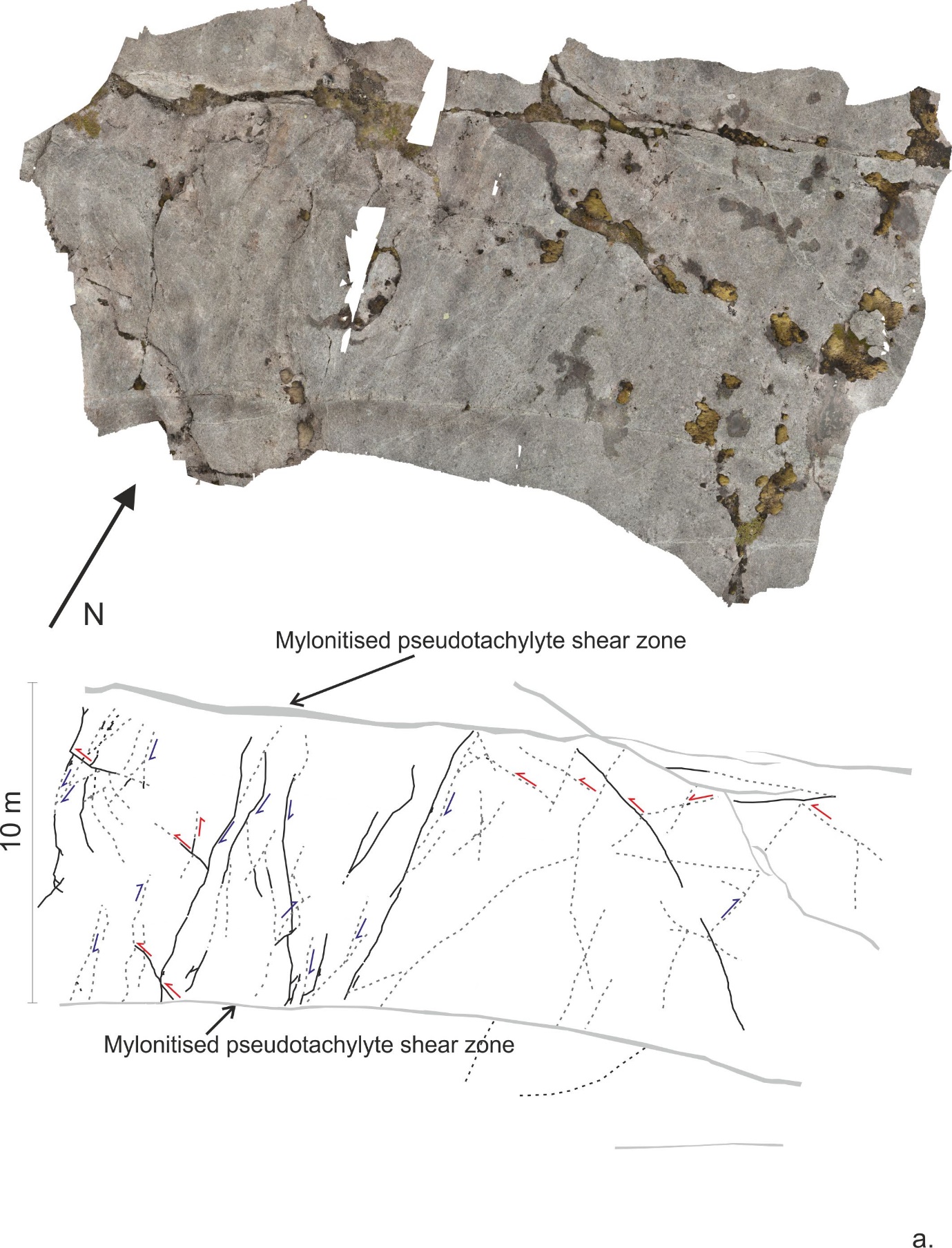


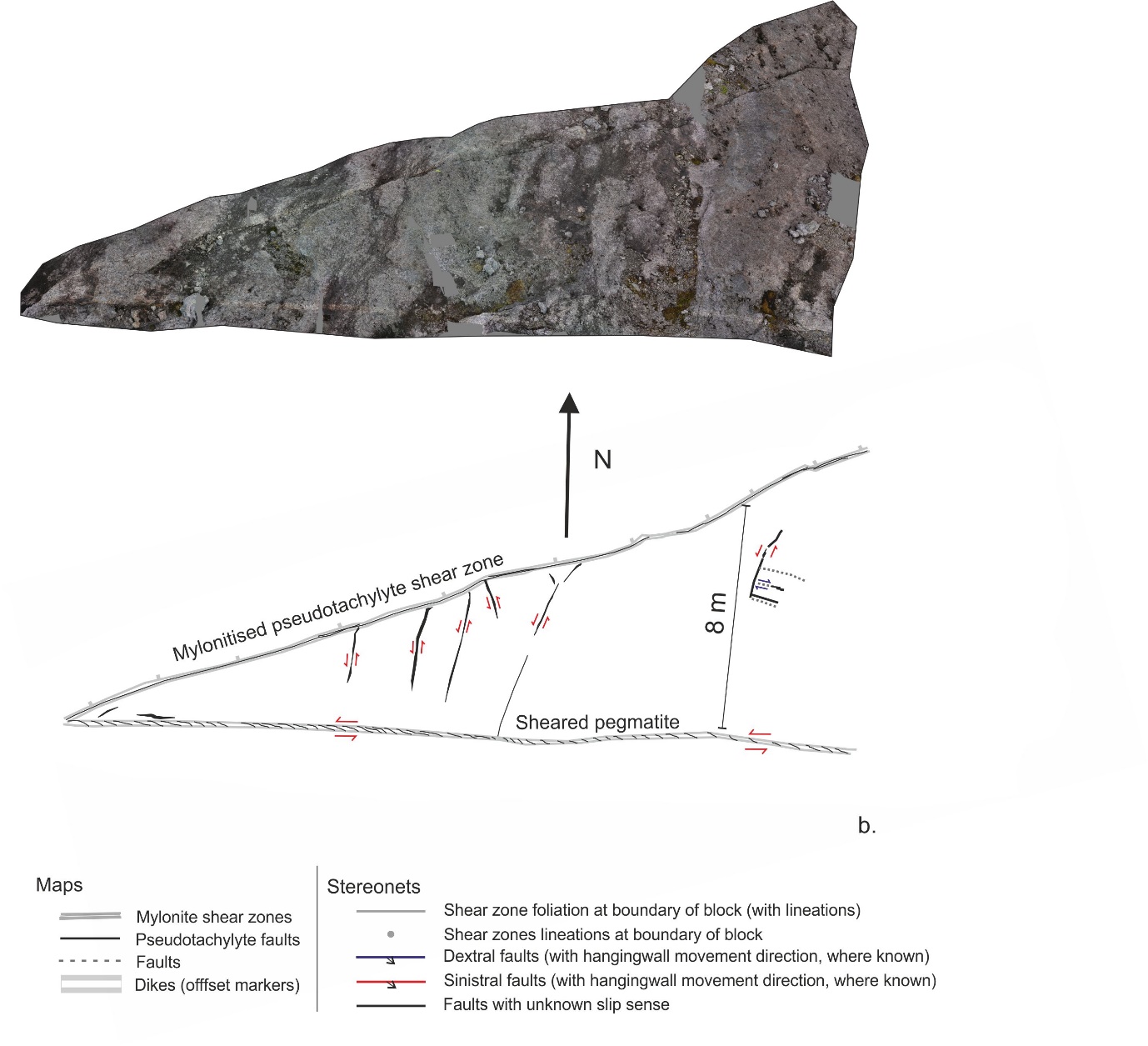


**Supplementary Figure 4:** Separate photomap and sketch maps from **a.** Fig. 3a and **b**. Fig. 3b.


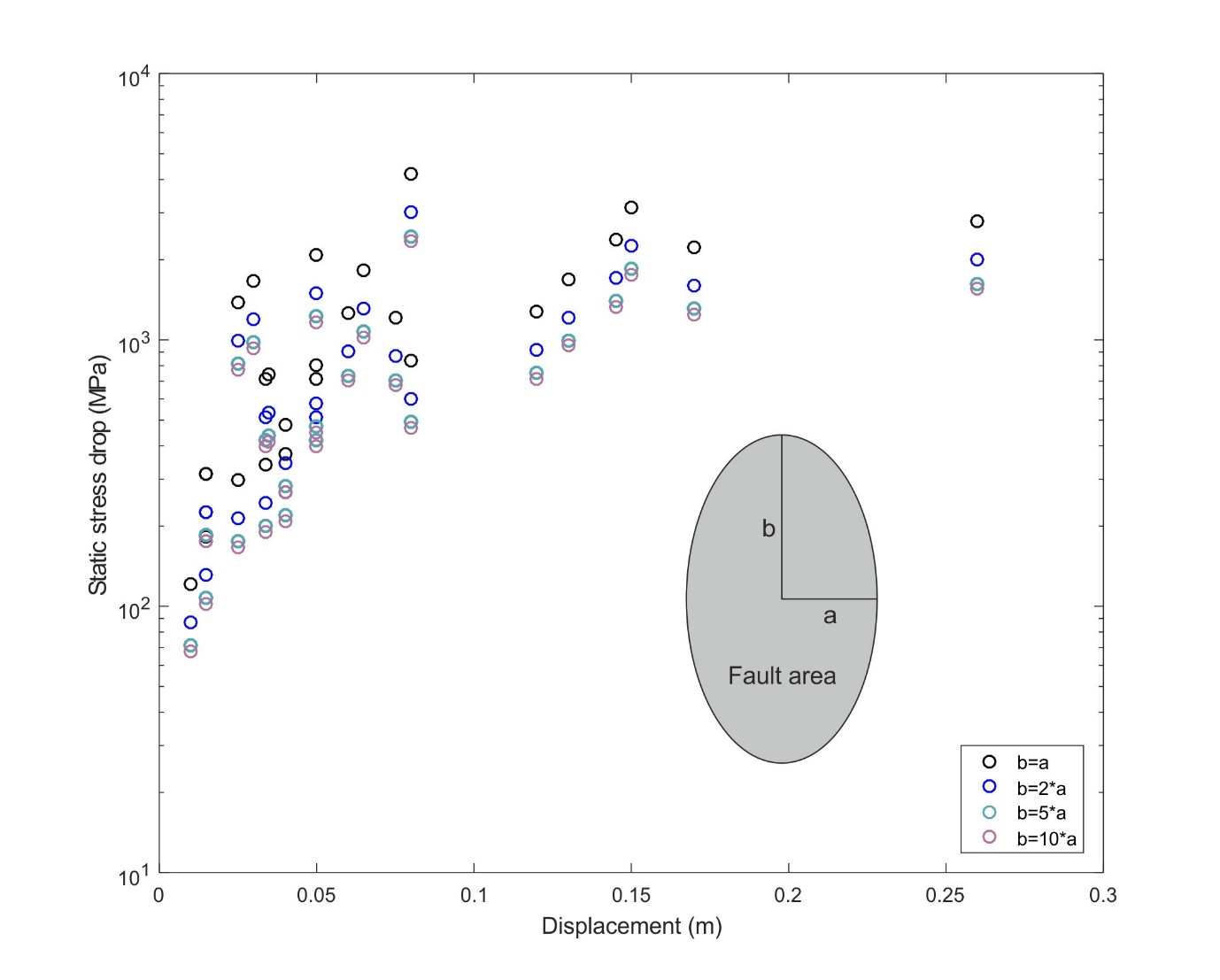


**Supplementary Figure 5:** Variation in static stress drops (plotted against fault slip displacement) of internal pseudotachylyte-bearing faults with varying elliptical fault geometry. The semi-minor axis is designated ‘a’ and the semi-major axis ‘b’. Circular faults (a = b) are shown alongside faults with increasing aspect ratio (b = 2a, b = 5a) up to a maximum aspect ratio of ten (b = 10a).

Supplementary references

1. Steltenpohl, M. G., Kassos, G., Andresen, A., Rehnström, E. F. & Hames, W. E. Eclogitization and exhumation of Caledonian continental basement in Lofoten, North Norway. *Geosph.* **7**, 202–218 (2011).
